# What will happen when thermoresponsive poly(*N*-isopropylacrylamide) is tethered on poly(ionic liquid)s?†

**DOI:** 10.1039/c9ra01849b

**Published:** 2019-04-26

**Authors:** Guangmei Luo, Yakun Guo, Chonggao Liu, Guang Han, Xiaodong Ma, Wangqing Zhang

**Affiliations:** Key Laboratory of Functional Polymer Materials of the Ministry of Education, Institute of Polymer Chemistry, College of Chemistry, Nankai University Tianjin 300071 China wqzhang@nankai.edu.cn +86-22-23503510; State Key Laboratory of Special Functional Waterproof Materials, Beijing Oriental Yuhong Waterproof Technology Co., Ltd Beijing 100123 China hanguang@yuhong.com.cn; School of Energy and Environmental Engineering, Hebei University of Technology Tianjin 300401 China; Collaborative Innovation Center of Chemical Science and Engineering (Tianjin), Nankai University Tianjin 300071 China

## Abstract

The thermoresponsive ionic liquid diblock copolymer of poly[1-(4-vinylbenzyl)-3-methylimidazolium tetrafluoroborate]-*block*-poly(*N*-isopropylacrylamide) (P[VBMI][BF_4_]-*b*-PNIPAM) containing a hydrophilic poly(ionic liquid) block of P[VBMI][BF_4_] is prepared by sequential reversible addition–fragmentation chain transfer (RAFT) polymerization. This P[VBMI][BF_4_]-*b*-PNIPAM exhibits an abnormal thermoresponsive phase transition at a temperature above the phase transition temperature (PTT) of the PNIPAM block. For P[VBMI][BF_4_]-*b*-PNIPAM including a short P[VBMI][BF_4_] block, its aqueous solution becomes turbid at a temperature above the PTT of the thermoresponsive PNIPAM block, whereas for P[VBMI][BF_4_]-*b*-PNIPAM containing a relatively long P[VBMI][BF_4_] block even in the case of a relatively long PNIPAM block, the aqueous solution remains transparent at a temperature far above the PTT of the PNIPAM block, although a soluble-to-insoluble phase transition of the PINIPAM block is confirmed by dynamic light scattering (DLS) analysis and variable temperature ^1^H NMR analysis. The reason that P[VBMI][BF_4_]-*b*-PNIPAM exhibits an abnormal thermoresponse is discussed and ascribed to the highly hydrophilic and charged poly(ionic liquid) block of P[VBMI][BF_4_] leading to the formation of small-sized micelles at a temperature above the PTT.

## Abstract 2

A thermoresponsive ionic liquid diblock copolymer shows abnormal thermoresponse, and its aqueous solution remains transparent at the phase transition temperature.

## Graphical abstract

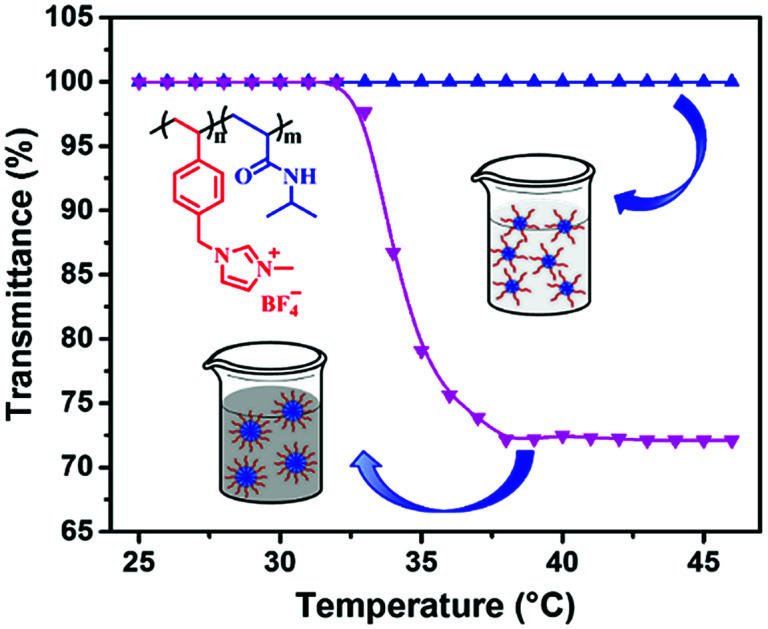

## Introduction

Polymerized ionic liquids or poly(ionic liquid)s (PILs), a subclass of polyelectrolytes with ionic moieties as the repeating unit, have attracted much attention due to their combination of properties from both ionic moieties and neutral polymers.^1^ PILs exhibit unique properties compared to common neutral polymers, such as high ion conductivity, chemical and thermal stability and tunable solubility, which endow them with wide applications.^2^ For example, PILs have been used in CO_2_ capture and stabilization of nanomaterials, and as components of electrochemical devices and a binder for lithium-ion batteries.^2^ Generally, there are two strategies to prepare PILs. The first one is *via* post-modification of neutral polymers, which is usually achieved by incorporation of ionic liquid moieties into the appending groups of neutral polymers.^3–7^ However, this method suffers from the nonquantitative metathesis reactions and therefore the polymer is usually contaminated by a tiny fraction of halides.^6^ The second approach is *via* direct polymerization of ionic liquid monomers through conventional free radical polymerization,^8–12^ controlled radical polymerization (CRP)^13–27^ and polycondensation,^28,29^ by which major PILs have been prepared. Of all these syntheses, controlled radical polymerizations (CRPs) including atom transfer radical polymerization (ATRP),^13–16^ reversible addition–fragmentation chain-transfer (RAFT) polymerization^17–26^ and cobalt-mediated radical polymerization (CMRP)^27^ have been applied to polymerization of imidazolium-based ionic liquid monomers containing the Br^−^, BF_4_^−^, PF_6_^−^ and Tf_2_N^−^ counterions to afford PILs with controlled molecular weight and narrow molecular weight distribution.

Thermoresponsive polymers represent an important class of stimuli-responsive materials, which undergo reversible phase transition at the lower critical solution temperature (LCST) or the upper critical solution temperature (UCST).^30,31^ For an aqueous solution of LCST-type thermoresponsive polymers, it exhibits a typical character of becoming turbid at temperature above LCST *via* soluble-to-insoluble phase transition.^30^ To date, thermoresponsive polymers such as *N*-substituted poly(meth)acrylamides,^32,33^*N*-alkyl-substituted poly(aminoethyl methacrylate)s,^34–36^ poly[oligo(ethylene glycol)(meth)acrylate]s,^37,38^ poly(2-alkyl-2-oxazoline)s,^39,40^ poly(vinyl methyl ester)s^41,42^ and polypeptides^43,44^ have been verified to undergo soluble-to-insoluble phase transition in aqueous solution at temperature above the phase transition temperature (PTT). Among them, poly(*N*-isopropylacrylamide) (PNIPAM),^45–53^ which has an LCST around 32 °C in water, is possibly the most widely studied thermoresponsive polymer.

Recently, various ionic liquid block copolymers (ILBCs) have been prepared by RAFT polymerization,^17–26^ and this RAFT technique affords advantages including good control in a broad range of monomers, metal-free formulation and relatively easy implementation just as conventional radical polymerization.^54^ Combination of PNIPAM and PILs *via* RAFT has attracted much attention, since it endows PILs with thermoresponse.^21–26^ For example, Mori and co-workers prepared well-defined ILBC of PNIPAM-*b*-poly(EtOEVI-Br) containing a long hydrophilic poly(EtOEVI-Br) block and found that the PTT of PNIPAM-*b*-poly(EtOEVI-Br) was higher than that of PNIPAM and no detectable micelle formation was observed.^23^ Yuan and co-workers synthesized a series of ILBCs containing PNIPAM and vinyl imidazole-based blocks, and they observed that the PTT of the block copolymers increased slightly compared to the PNIPAM homopolymer, and they formed micelles or other kinds of aggregates at temperature above PTT.^24^ Karjalainen and coworkers synthesized amphiphilic ILBCs including an insoluble PIL block and a thermoresponsive PNIPAM block by RAFT polymerization, and it was revealed that micelles were formed at the case of a long PNIPAM block, *i.e.* PIL_24_-PNIPAM_88_, although the phase transition was very weak. Furthermore, these micelles could not convert into large aggregates upon heating.^25^

In this contribution, thermoresponsive ILBCs of poly[1-(4-vinylbenzyl)-3-methylimidazolium tetrafluoroborate]-*block*-poly(*N*-isopropylacrylamide) (P[VBMI][BF_4_]-*b*-PNIPAM) containing a hydrophilic poly(ionic liquid) segment of P[VBMI][BF_4_] were prepared by sequential RAFT polymerization. The thermoresponsive behavior of double hydrophilic P[VBMI][BF_4_]-*b*-PNIPAM with different degree of polymerization (DP) of the P[VBMI][BF_4_] and PNIPAM blocks was carefully explored by turbidity analysis, dynamic light scattering (DLS) analysis and variable temperature ^1^H NMR analysis. It is found that inserting a hydrophilic P[VBMI][BF_4_] block leads to an increasing PPT of P[VBMI][BF_4_]-*b*-PNIPAM. But interestingly, P[VBMI][BF_4_]-*b*-PNIPAM containing a relatively long P[VBMI][BF_4_] block, *e.g.*, P[VBMI][BF_4_]_76_-*b*-PNIPAM_226_, exhibits an abnormal thermoresponse. That is, the aqueous solution of P[VBMI][BF_4_]-*b*-PNIPAM does not become turbid even at temperature above PTT of the PNIPAM block, although the soluble-to-insoluble phase transition of the PINIPAM block is confirmed by dynamic light scattering (DLS) analysis and variable temperature ^1^H NMR analysis. This abnormal thermoresponse of P[VBMI][BF_4_]-*b*-PNIPAM is different from those of ILBCs and is ascribed to the formation of small-sized micelles.

## Experimental

### Materials

The chemical reagents including chloromethylstyrene (CMS, >97%, Alfa), *N*-methylimidazole (98%, Tianjin Yichuangcheng Chemical Company), sodium tetrafluoroborate (NaBF_4_, >99%, Tianjin Chemical Company) and hydroquinone (>98%, Tianjin Chemical Company) were used as received. The monomer of *N*-isopropylacrylamide (NIPAM, >99%, Acros Organics) was purified by recrystallization in an acetone/*n*-hexane mixture (50/50 by volume). The ionic liquid monomer of 1-(4-vinylbenzyl)-3-methylimidazolium tetrafluoroborate ([VBMI][BF_4_], seeing ^1^H NMR spectrum in Fig. S1†) was synthesized initially by nucleophilic substitution reaction of CMS with *N*-methylimidazole and then anion exchange reaction with sodium tetrafluoroborate.^8^ The initiator of 2,2′-azobis(isobutyronitrile) (AIBN, >98%, Tianjin Ruijinte Chemical Reagent) was recrystallized from ethanol prior to use. *S*-1-Dodecyl-*S*′-(α,α′-dimethyl-α′′-acetic acid)trithiocarbonate (DDMAT) was synthesized as discussed elsewhere.^55^ All the other reagents were analytic grade and used as received. Deionized water was used in the present study.

### Synthesis of P[VBMI][BF_4_] and P[VBMI][BF_4_]-*b*-PNIPAM

The homopolymers of P[VBMI][BF_4_] with different DPs (Table 1) were synthesized in the methanol/water mixture (80/20 by weight) by solution RAFT polymerization as discussed in our previous study.^17^ The experimental details can be found in ESI.† The ILBCs of P[VBMI][BF_4_]-*b*-PNIPAM (Table 1) were synthesized *via* RAFT polymerization of NIPAM in the methanol/water mixture (80/20 by weight) employing the prepared P[VBMI][BF_4_] as macromolecular RAFT agent (macro-RAFT) under [NIPAM]_0_ : [P[VBMI][BF_4_]]_0_ : [AIBN]_0_ = 360–1200 : 3 : 1. Herein, a typical polymerization to synthesize P[VBMI][BF_4_]_76_-*b*-PNIPAM_226_ was introduced. Into a Schlenk flask, P[VBMI][BF_4_]_76_ (0.400 g, 0.018 mmol), NIPAM (0.513 g, 4.55 mmol), AIBN (0.996 mg, 0.0060 mmol) dissolved in the 80/20 methanol/water mixture (3.65 g), and the internal standard 1,3,5-trioxane (0.0553 g, 0.614 mmol) for ^1^H NMR analysis were weighed. The solution was initially degassed with nitrogen with the help of iced water and then the flask content was immersed into a preheated oil bath at 70 °C. After 3 h, polymerization was quenched by rapid cooling to 0 °C, and 90% monomer conversion was determined by ^1^H NMR analysis according to eqn (S1)† by comparing the integral area of *δ* = 6.25 ppm corresponding to the residual monomer and that of *δ* = 5.12 ppm corresponding to the 1,3,5-trioxane internal standard. The synthesized P[VBMI][BF_4_]_76_-*b*-PNIPAM_226_ was precipitated in diethyl ether, the collected precipitate was washed three times with diethyl ether, and finally dried under vacuum at room temperature. By changing the molar ratio of [NIPAM]_0_/[P[VBMI][BF_4_]]_0_ or changing the molecular weight of P[VBMI][BF_4_], P[VBMI][BF_4_]-*b*-PNIPAM with different DPs as shown in Table 1 was prepared.

**Table 1:** Summaries of the synthesized P[VBMI][BF_4_] and P[VBMI][BF_4_]-*b*-PNIPAM

Polymer^a^		[M]_0_ : [CTA]_0_ : [AIBN]_0_	Time (h)	Conv.^b^ (%)	*M*_n,th_ c (kg mol^−1^)	*M*_n,NMR_ d (kg mol^−1^)	*M*_n,GPC_ e (kg mol^−1^)	*Ð* f
Homopolymer	N_223_	1000 : 4 : 1	2	89	25.6	24.8	21.5	1.16
	PIL_18_	60 : 3 : 1	6	91	5.5	4.9	7.8	1.13
	PIL_37_	120 : 3 : 1	6	92	10.9	10.3	11.9	1.12
	PIL_56_	180 : 3 : 1	6	93	16.3	15.6	12.7	1.15
	PIL_76_	270 : 3 : 1	6	84	22.0	21.5	13.9	1.16
First group	I_18_N_239_	750 : 3 : 1	3	96	32.5	35.4	25.8	1.18
	I_37_N_231_	750 : 3 : 1	3	92	37.0	37.9	23.9	1.14
	I_56_N_221_	750 : 3 : 1	3	88	41.3	40.6	17.7	1.26
Second group	I_76_N_226_	750 : 3 : 1	3	90	47.6	46.5	23.9	1.11
	I_76_N_92_	360 : 3 : 1	3	77	32.4	32.5	11.0	1.07
	I_76_N_364_	1200 : 3 : 1	3	91	63.2	58.7	30.6	1.14

a I represents the P[VBMI][BF_4_] block and N represents the PNIPAM block.

b Monomer conversion determined by ^1^H NMR analysis.

c Theoretical molecular weight determined by monomer conversion.

d Molecular weight determined by ^1^H NMR analysis.

e Molecular weight determined by GPC analysis.

f Dispersity (*Ð*) determined by GPC analysis.

### Synthesis of PNIPAM homopolymer

Herein, the synthesis of the reference homopolymer of PNIPAM_223_ is introduced. Into a 25 mL Schlenk flask with a magnetic bar, NIPAM (3.00 g, 26.5 mmol), DDMAT (38.8 mg, 0.106 mmol), AIBN (4.4 mg, 0.0267 mmol) and 1,4-dioxane (12.0 g) were added. The flask content was degassed and then the flask was immersed in a preheated oil bath at 70 °C for 2 h. The polymerization was quenched by rapid cooling upon immersion of the flask in iced water. The monomer conversion of 89% was determined by ^1^H NMR analysis, which was calculated according to eqn S(2)† by comparing the integral area of *δ* = 6.00–6.10 ppm corresponding to the residual monomer and that of *δ* = 1.05–1.22 ppm corresponding to the methyl in NIPAM and PNIPAM. The synthesized polymer was purified by three precipitation–filtration cycles in iced diethyl ether, and then dried under vacuum at room temperature overnight, and characterized by GPC (Fig. S2†).

### Characterization

The ^1^H NMR analysis was performed on a Bruker Avance III 400 MHz NMR spectrometer using CDCl_3_, D_2_O or DMSO-*d*_6_ as solvent. Molecular weight and dispersity (*Ð*, *Ð* = *M*_w_/*M*_n_) were determined by gel permeation chromatography (GPC) equipped with a DAWN HELEOS 8 light scattering photometer, a ViscoStar viscometer, an Optilab rEX interferometric refractometer and 3 Mz-Gel SD plus 10 μm columns, in which DMF containing 0.01 mol L^−1^ NaBF_4_ and 0.01 mol L^−1^*N*-methylimidazole was used as eluent at flow rate of 0.8 mL min^−1^ at 50 °C and the narrow-polydispersity poly(methyl methacrylate) (PMMA) was used as calibration standard. Turbidity analysis was performed on a Varian 100 UV-vis spectrophotometer equipped with a thermo-regulator (±0.1 °C). In the turbidity analysis, 2.0 wt% polymer aqueous solution was heated at 1 °C min^−1^, transmittance at 500 nm was recorded after the temperature was kept for 5 min, and the LCST or phase transition temperature (PTT) was determined at the half of the maximal and minimal transmittance. Dynamic light scattering (DLS) analysis was performed on a laser light scattering spectrometer (BI-200SM) equipped with a digital correlator (BI-10000AT) at 636 nm. Before DLS analysis, the samples of 0.5 wt% polymer aqueous solution were filtered through a 0.45 μm Millipore filter to remove dust. The transmission electron microscopy (TEM) observation was performed using a Tecnai G2 F20 electron microscope at an acceleration voltage of 200 kV. In TEM sampling, 0.5 wt% polymer aqueous solution (1 mL) was heated and kept at 50 °C, then 1 wt% aqueous solution of phosphotungstic acid (PTA, 1 mL) at 50 °C was added, and the mixture was kept at 50 °C for about 5 min, then a small drop of the diluted aqueous solution was deposited onto a piece of copper grid pre-heated at 50 °C until the solvent was fully evaporated, and finally the samples were checked by TEM. Average size of formed micelles was obtained by analyzing more than 100 nanoparticles using the ImageJ software.

## Results and discussion

### Synthesis of P[VBMI][BF_4_] and P[VBMI][BF_4_]-*b*-PNIPAM

**Scheme 1:**
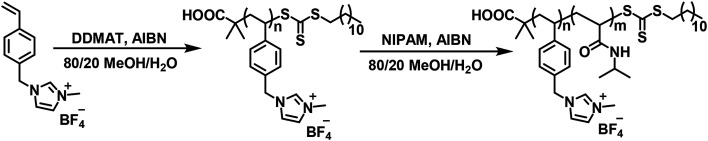
The schematic synthesis of P[VBMI][BF_4_] and P[VBMI][BF_4_]-*b*-PNIPAM.

**Fig. 1:**
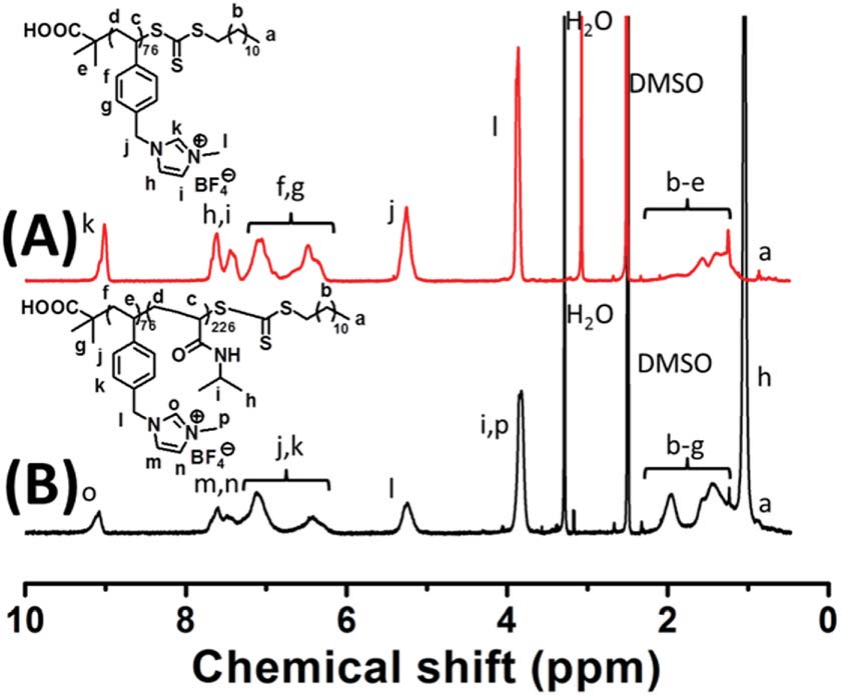
^1^H NMR spectra of P[VBMI][BF_4_]_76_ (A) and P[VBMI][BF_4_]_76_-*b*-PNIPAM_226_ (B), in which DMSO-*d*_6_ is used as solvent.

Scheme 1 outlines the synthesis of P[VBMI][BF_4_]-*b*-PNIPAM by sequential RAFT polymerization, in which the initial synthesis of P[VBMI][BF_4_] by solution RAFT polymerization under [[VBMI][BF_4_]]_0_ : [DDMAT]_0_ : [AIBN]_0_ = 60–270 : 3 : 1 and the subsequent synthesis of P[VBMI][BF_4_]-*b*-PNIPAM by solution RAFT polymerization under [NIPAM]_0_ : [P[VBMI][BF_4_]]_0_ : [AIBN]_0_ = 360–1200 : 3 : 1 are included. For briefness, P[VBMI][BF_4_]-*b*-PNIPAM is named I_*x*_N_*y*_, in which I and N represent the P[VBMI][BF_4_] and PNIPAM blocks and *x* and *y* represent the DPs, respectively. By varying the molar ratio of [VBMI][BF_4_]/DDMAT, four homopolymers of P[VBMI][BF_4_]_18_, P[VBMI][BF_4_]_37_, P[VBMI][BF_4_]_56_ and P[VBMI][BF_4_]_76_ with the theoretic molecular weight (*M*_n,th_) at 5.5, 10.9, 16.3, 22.0 kg mol^−1^ and the theoretical DP at 18, 37, 56, 76, in which *M*_n,th_ is calculated by the monomer conversion according to eqn S(3),† were synthesized at 80–90% monomer conversion. Fig. 1A shows the ^1^H NMR spectrum of the typical P[VBMI][BF_4_]_76_, in which the characteristic proton chemical shifts assigned to the P[VBMI][BF_4_] backbone and the RAFT terminal are clearly observed. By comparing the chemical shifts at 3.87 ppm (l in Fig. 1A) and 0.88 ppm (a in Fig. 1A), the molecular weight *M*_n,NMR_ of P[VBMI][BF_4_] is obtained according to eqn S(4).† The synthesized P[VBMI][BF_4_] is further characterized by GPC, from which the molecular weight *M*_n,GPC_ and *Ð* are obtained. See the characterization of P[VBMI][BF_4_] in Table 1 and also in our previous manuscript.^17^

**Fig. 2:**
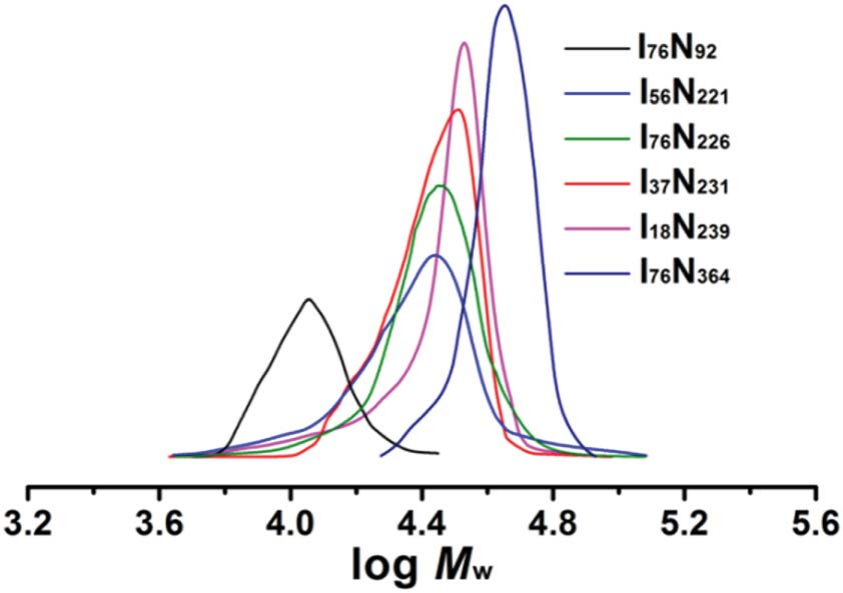
GPC traces of P[VBMI][BF_4_]-*b*-PNIPAM.

The ILBCs of P[VBMI][BF_4_]-*b*-PNIPAM were prepared by using P[VBMI][BF_4_] as macro-RAFT agent under [NIPAM]_0_ : [P[VBMI][BF_4_]]_0_ : [AIBN]_0_ = 360–1200 : 3 : 1 at 70 °C. After 3 h polymerization, around 90% monomer conversion was achieved, and ILBCs with different DPs were prepared. The ILBCs were characterized by ^1^H NMR analysis and GPC analysis. Fig. 1B shows the ^1^H NMR spectrum of the typical I_76_N_226_, in which the characteristic proton signals of the P[VBMI][BF_4_] block and PNIPAM block are clearly observed. The molecular weight *M*_n,NMR_ at 46.5 kg mol^−1^, which is calculated following eqn S(5)† by comparing the signal at *δ* = 9.1 ppm (o in Fig. 1B) corresponding to the imidazole ring with the signal at *δ* = 3.84 ppm (i and p in Fig. 1B) corresponding to the methyl and methane connected to the N atom, is close to the theoretic molecular weight *M*_n,th_ at 47.6 kg mol^−1^ calculated following eqn S(3).† As summarized in Table 1, *M*_n,NMR_ of P[VBMI][BF_4_]-*b*-PNIPAM is very close to *M*_n,th_, suggesting good control on polymer molecular weight in the present RAFT polymerization. Fig. 2 shows the GPC traces of P[VBMI][BF_4_]-*b*-PNIPAM, from which molecular weight *M*_n,GPC_ and *Ð* are obtained and results are summarized in Table 1. All P[VBMI][BF_4_]-*b*-PNIPAM ILBCs have narrow molecular weight distribution as indicated by *Ð* below 1.3. However, *M*_n,GPC_ is generally smaller than *M*_n,th_ and *M*_n,NMR_, and this is possibly due to the PMMA standards used in the GPC analysis, since PILs have different hydrodynamic volumes compared to neutral polymer standards.^56^ Besides, the strong interaction between ionic liquid P[VBMI][BF_4_] block and the stationary phases of the GPC columns may lead to the underestimation of *M*_n,GPC_.^56^

The synthesis of ILBCs was also tried by first polymerizing NIPAM followed by ionic liquid monomer of [VBMI][BF_4_]. It was found that relatively low monomer conversion of [VBMI][BF_4_] was obtained in the presence of PNIPAM macro-RAFT. This suggests that the polymerization activity of PNIPAM macro-RAFT is lower than that of P[VBMI][BF_4_] macro-RAFT.

### Thermoresponse of P[VBMI][BF_4_]-*b*-PNIPAM in water

The thermoresponsive behavior of P[VBMI][BF_4_]-*b*-PNIPAM in water is checked. As indicated in our previous study,^17^ P[VBMI][BF_4_] is soluble in water and PNIPAM exhibits an LCST around 32 °C in water, and therefore P[VBMI][BF_4_]-*b*-PNIPAM is soluble in water at room temperature below LCST of the PNIPAM block. This makes it easy to prepare the P[VBMI][BF_4_]-*b*-PNIPAM aqueous solution just by dissolving polymers in water at room temperature.

**Fig. 3:**
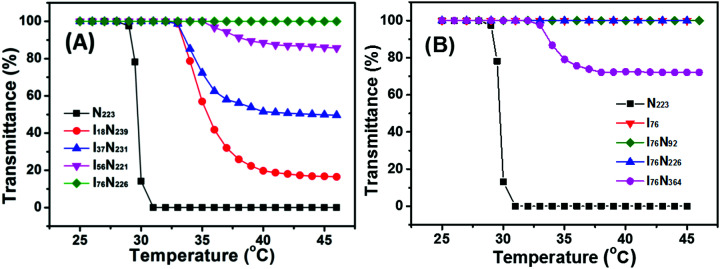
The temperature-dependent transmittance of P[VBMI][BF_4_]-*b*-PNIPAM with different DP of P[VBMI][BF_4_] block (A) and different DP of PNIPAM block (B), in which the polymer concentration is 2.0 wt%.

To check the thermoresponse of P[VBMI][BF_4_]-*b*-PNIPAM, ILBCs are divided into groups, one having a similar DP around 220 of the PNIPAM block but different DP of the P[VBMI][BF_4_] block and the other having a constant DP of the P[VBMI][BF_4_] block at 76 but different DP of the PNIPAM block. The DP of PNIPAM is set at about 220 and therefore we can evaluate the effect of the length of hydrophilic P[VBMI][BF_4_] block on thermoresponse of P[VBMI][BF_4_]-*b*-PNIPAM. Fig. 3A shows the temperature-dependent transmittance of the 2.0 wt% aqueous solution of the first group ILBCs. It indicates that the LCST-type phase transition is strongly correlated to the poly(ionic liquid) block of P[VBMI][BF_4_]. First, the PTT of P[VBMI][BF_4_]-*b*-PNIPAM increases in comparison with the PNIPAM homopolymer till the aqueous solution of I_76_N_226_ having the longest P[VBMI][BF_4_] block keeps transparent even at a temperature far above the LCST of the PNIPAM block. For instance, the PNIPAM_223_ homopolymer exhibits an LCST at 31 °C, and the PTT of I_18_N_239_, I_37_N_231_ and I_56_N_221_ increases to 35.0 °C, 35.0 °C and 37.0 °C, respectively. It is known that inserting a hydrophilic polymer segment into a thermoresponsive polymer usually leads to an increasing PTT of thermoresponsive copolymers.^22–24,30,51^ Clearly, the PTTs of P[VBMI][BF_4_]-*b*-PNIPAM ILBCs follow the similar trend. However, it is very surprising that the aqueous solution of I_76_N_226_ having such a long PNIPAM_226_ block keeps transparent, which will be further discussed subsequently. Second, with the DP of the P[VBMI][BF_4_] block increasing, the P[VBMI][BF_4_]-*b*-PNIPAM aqueous solution becomes less turbid as indicated by the increasing transmittance shown in Fig. 3A, suggesting that P[VBMI][BF_4_]-*b*-PNIPAM having a long poly(ionic liquid) block of P[VBMI][BF_4_] tends to form small micelles in water at temperature above PTT, which is as similar as those of amphiphilic block copolymers or double hydrophilic block copolymers containing a thermoresponsive block.^57–62^ To explore the unique thermoresponse of P[VBMI][BF_4_]_76_-*b*-PNIPAM having a long P[VBMI][BF_4_] block, the thermoresponse of the second group ILBCs is checked and the effect of the length of PNIPAM on thermoresponse is evaluated. As shown in Fig. 3B, just P[VBMI][BF_4_]_76_-*b*-PNIPAM_364_ having a very long PNIPAM_364_ block becomes turbid at temperature above PTT around 34 °C, whereas other I_76_N_*y*_ samples, *e.g.*, P[VBMI][BF_4_]_76_, P[VBMI][BF_4_]_76_-*b*-PNIPAM_92_ and P[VBMI][BF_4_]_76_-*b*-PNIPAM_226_, keep transparent even at the temperature far above LCST of the PNIPAM block, although the PNIPAM block, *e.g.*, PNIPAM_226_, is relatively long.

The turbidity analysis indicates an abnormal thermoresponse of P[VBMI][BF_4_]-*b*-PNIPAM including a long P[VBMI][BF_4_] block. To make sure that it is the P[VBMI][BF_4_] block leading to the abnormal thermoresponse, the aqueous solution containing equal molar of the PNIPAM_223_ and P[VBMI][BF_4_]_76_ homopolymers is checked. It is found that the aqueous solution becomes turbid at temperature above LCST of the PNIPAM_223_ homopolymer (Fig. S3†). This suggests that the tethered P[VBMI][BF_4_]_76_ block leads to the abnormal thermoresponse of P[VBMI][BF_4_]-*b*-PNIPAM.

**Fig. 4:**
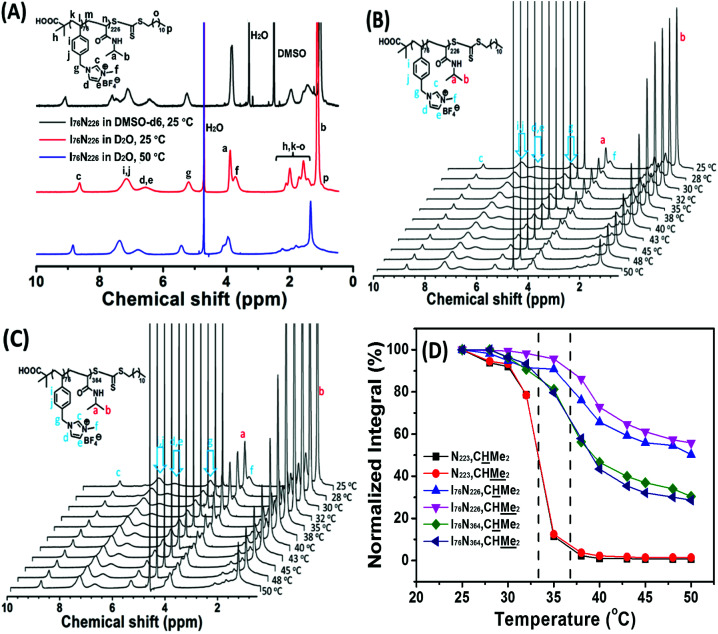
The ^1^H NMR spectra of I_76_N_226_ in DMSO-*d*_6_ and D_2_O with 2.0 wt% polymer concentration (A), the temperature-dependent ^1^H NMR spectra of I_76_N_226_ (B) and I_76_N_364_ (C) in D_2_O, and the temperature-dependent normalized integrals of the typical proton signals of N_223_, I_76_N_226_ and I_76_N_364_ (D) in D_2_O. Note: PTT is defined at 50% integral decrease.

Variable temperature ^1^H NMR analysis is a valid method to detect phase transition of thermoresponsive polymers.^63,64^ Herein, two typical ILBCs, *e.g.*, I_76_N_226_ keeping transparent at temperature above PTT of the PNIPAM block and I_76_N_364_ becoming slightly turbid at temperature above 34 °C, are investigated by variable temperature NMR analysis. Fig. 4A shows the ^1^H NMR spectra of 2.0 wt% I_76_N_226_ in D_2_O at 25 °C and 50 °C as well as in DMSO-*d*_6_ at 25 °C, in which all the signals are normalized by that at around 8.9 ppm (c, seeing the NMR spectra in Fig. 4A) assigned to the soluble poly(ionic liquid) block of P[VBMI][BF_4_]. It indicates that ^1^H NMR spectrum of I_76_N_226_ in D_2_O at 25 °C is very similar with that in DMSO-*d*_6_ at 25 °C although the chemical shifts are slightly different, indicating that I_76_N_226_ is molecularly soluble in D_2_O at 25 °C. At temperature of 50 °C, the signals assigned to the poly(ionic liquid) block of P[VBMI][BF_4_] almost keep constant (c, d, e, f, g, i, j and *etc.*), whereas the signals assigned to the PNIPAM block (a, b, m, n) are greatly weakened. This confirms that the soluble-to-insoluble phase transition of the PNIPAM_226_ block really occurs, although the aqueous solution keeps transparent. To further investigate the thermoresponse, the ^1^H NMR spectra of 2.0 wt% aqueous (D_2_O) solution of I_76_N_226_ (Fig. 4B and S4A†) and I_76_N_364_ (Fig. 4C and S4B†) as well as the reference PNIPAM_223_ homopolymer at temperature ranging from 25 °C (below PTT) to 50 °C (above PTT) are recorded and the two typical signals at 3.93 ppm (a, CHMe_2_) and 1.17 ppm (b, CHMe_2_) assigned to the PNIPAM block at different temperatures are summarized in Fig. 4D. As shown in Fig. 4D, the decreasing signals of a and b indicate the soluble-to-insoluble phase transition of the PNIPAM block in ILBCs of both I_76_N_226_ and I_76_N_364_. Similarly within turbidity analysis, the NMR-determined PTT of the PNIPAM block, 37 °C for both I_76_N_226_ and I_76_N_364_ and 33 °C for PNIPAM_223_, is defined at the 50% decrease of intensity. This PTT by ^1^H NMR is slightly higher than that by turbidity analysis, and the slight difference is possibly ascribed to the deuterated solvent.^33,65,66^ To evaluate the dehydration of PNIPAM in the phase transition, the fraction of dehydration, *p*, which is defined by eqn S(6),†^63^ is calculated by assuming *p* = 1 for the homopolymer of PNIPAM_223_ (*p* = 1 for PNIPAM_223_, *p* = 0.65 for I_76_N_364_ and *p* = 0.35 for I_76_N_226_). This suggests that the poly(ionic liquid) block of P[VBMI][BF_4_] decreases the dehydration of PNIPAM, and P[VBMI][BF_4_]-*b*-PNIPAM having a shorter PNIPAM block is less dehydrated. This less dehydration of the PNIPAM block in P[VBMI][BF_4_]-*b*-PNIPAM having a long P[VBMI][BF_4_] block means less turbidity of the aqueous solution.

**Fig. 5:**
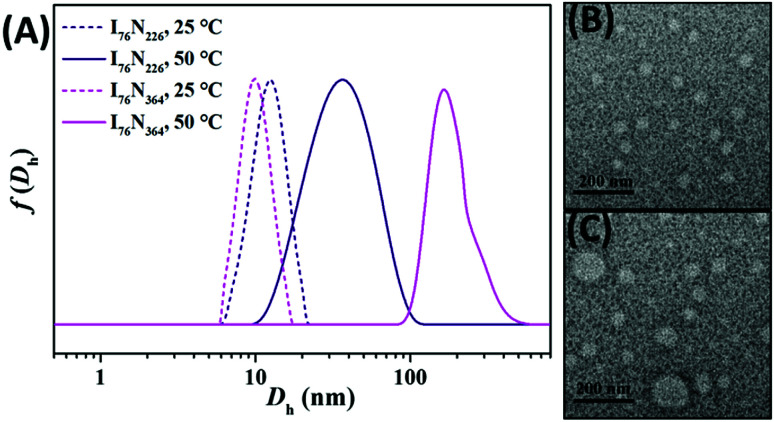
Hydrodynamic diameter distribution *f*(*D*_h_) of 0.5 wt% aqueous solution of I_76_N_226_ and I_76_N_364_ at 25 °C and 50 °C (A), the TEM images of micelles of I_76_N_226_ (B) and I_76_N_364_ formed in water at 50 °C (C). Note: the micelles were dried onto a copper grid and stained by PTA.

The thermoresponse of two typical ILBCs of I_76_N_226_ and I_76_N_364_ is further investigated by DLS analysis. Note: seeing Fig. S5† for P[VBMI][BF_4_]-*b*-PNIPAM with different DPs of the P[VMBI][BF_4_] block. As shown in Fig. 5A, I_76_N_226_ and I_76_N_364_ are soluble in water at 25 °C below PTT as indicated by the hydrodynamic diameter *D*_h_ below or around 10 nm. At temperature of 50 °C above PTT, I_76_N_226_ forms micelles with *D*_h_ at 38 nm and I_76_N_364_ forms micelles with *D*_h_ at 164 nm. The formation of micelles is further confirmed by TEM (Fig. 5B and C), in which 35–50 nm nanoparticles and 45–110 nm nanoparticles are observed. This confirms that the thermoresponsive phase transition of I_76_N_226_ and I_76_N_364_ at temperature above PTT. Since the I_76_N_226_ micelles are relatively small, the aqueous solution at temperature above PTT keeps transparent. It is thought that the poly(ionic liquid) block of P[VBMI][BF_4_] is highly hydrophilic and charged and therefore is highly repulsive. This character leads to small-sized micelles and the abnormal transparent solution at temperature above PTT of the PNIPAM block.

**Scheme 2:**
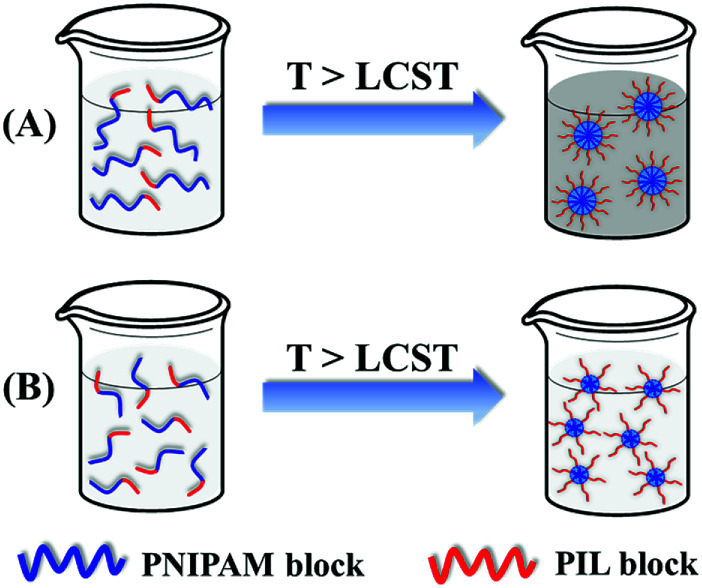
Schematic illustration of the thermoresponse of I_76_N_364_ having a very long PNIPAM block (A) and I_76_N_226_ in which the PNIPAM block is not long enough (B).

Summarily, the ILBCs of double hydrophilic P[VBMI][BF_4_]-*b*-PNIPAM having a relatively long P[VBMI][BF_4_] block exhibit abnormal thermoresponse. As shown in Scheme 2, it forms large-sized micelles at the case of a very long PNIPAM block through soluble-to-insoluble phase transition at temperature above PTT of the thermoresponsive PNIPAM block and the aqueous solution becomes turbid, otherwise just small-sized micelles are formed and the aqueous solution keeps transparent. This transparent aqueous solution of P[VBMI][BF_4_]-*b*-PNIPAM at temperature above PTT seems abnormal, and the phenomenon is ascribed to the highly hydrophilic and charged poly(ionic liquid) block of P[VBMI][BF_4_].

## Conclusions

Thermoresponsive ILBCs of P[VBMI][BF_4_]-*b*-PNIPAM containing a hydrophilic poly(ionic liquid) block of P[VBMI][BF_4_] were synthesized by RAFT polymerization. The synthesized P[VBMI][BF_4_]-*b*-PNIPAM has well-defined molecular weight and narrow molecular weight distribution. The thermoresponsive behavior P[VBMI][BF_4_]-*b*-PNIPAM was explored by turbidity analysis, DLS and variable temperature ^1^H NMR. Turbidity analysis indicates, when the P[VBMI][BF_4_] block is not long enough, the aqueous solution of P[VBMI][BF_4_]-*b*-PNIPAM becomes turbid at temperature above PTT, and PTT slightly increases with the increasing DP of the P[VBMI][BF_4_] block. Interestingly, for P[VBMI][BF_4_]-*b*-PNIPAM containing a relatively long P[VBMI][BF_4_] block and a relatively long PNIPAM block, *e.g.*, P[VBMI][BF_4_]_76_-*b*-PNIPAM_226_, the aqueous solution of P[VBMI][BF_4_]-*b*-PNIPAM keeps transparent at temperature above LCST of the PNIPAM block. Dynamic light scattering (DLS) analysis and variable temperature ^1^H NMR analysis indicates a soluble-to-insoluble phase transition of the PNIPAM block at temperature above PTT, although the aqueous solution of P[VBMI][BF_4_]-*b*-PNIPAM keeps transparent. The thermoresponse of P[VBMI][BF_4_]-*b*-PNIPAM seems a little abnormal in comparison with those general thermoresponsive block copolymers containing a hydrophilic block, and this abnormal phenomenon is ascribed to the highly hydrophilic and charged poly(ionic liquid) block of P[VBMI][BF_4_]. This P[VBMI][BF_4_]-*b*-PNIPAM tends to form small-sized micelles and keeps transparent at temperature above PTT of the PNIPAM block, and is anticipated to have promising application.

## Conflicts of interest

There are no conflicts to declare.

## Supplementary Material

RA-009-C9RA01849B-s001
