# Supplementary material for: What will happen when thermoresponsive poly(N-isopropylacrylamide) is tethered on poly(ionic liquid)s?
Source: RSC Adv. 2019 Apr 26;9(23):12936–43. doi: 10.1039/c9ra01849b (PMC9063810; doi:10.1039/c9ra01849b)
Supplement: RA-009-C9RA01849B-s001 [file RA-009-C9RA01849B-s001.pdf]

Electronic Supplementary Material (ESI) for RSC Advances.
This journal is © The Royal Society of Chemistry 2019

Supporting Information

What will happen when thermoresponsive poly(*N*-isopropylacrylamide) is tethered on poly(ionic liquid)s?

Guangmei Luo, Yakun Guo, Chonggao Liu, Guang Han*, Xiaodong Ma, and Wangqing Zhang*

Key Laboratory of Functional Polymer Materials of the Ministry of Education, Collaborative Innovation Center of Chemical Science and Engineering (Tianjin), Institute of Polymer Chemistry, Nankai University, Tianjin 300071, China.

State Key Laboratory of Special Functional Waterproof Materials, Beijing Oriental Yuhong Waterproof Technology Co., Ltd, Beijing 100123, China.

School of Energy and Environmental Engineering, Hebei University of Technology, Tianjin 300401, China.

*To whom correspondence should be addressed. E-mail: hanguang@yuhong.com.cn, wqzhang@nankai.edu.cn, Tel: 86-22-23509794, Fax: 86-22-23503510.

1. Synthesis of 1-(4-vinylbenzyl)-3-methylimidazolium tetrafluoroborate

The ionic liquid monomer of 1-(4-vinylbenzyl)-3-methylimidazolium tetrafluoroborate ([VBMI][BF_4]) was synthesized initially by the nucleophilic substitution reaction of CMS with *N*-methylimidazole and then the anion exchange reaction with sodium tetrafluoroborate. Into a flask, *N*-methylimidazole (16.40 g, 0.20 mol), CMS (30.40 g, 0.20 mol), hydroquinone (0.10 g) and acetone (100 mL) were added. The flask content was stirred at 40 ℃ for 24 h with magnetic stirring under nitrogen atmosphere. The resultant mixture was added dropwise into diethyl ether, and then kept at -10 ℃ overnight to deposit the imidazolium salt of 1-(4-vinylbenzyl)-3-methylimidazolium chloride ([VBMI]Cl). The precipitate of [VBMI]Cl was collected, washed with ethyl ether (500 mL × 2), and dried overnight under vacuum at room temperature to give a transparent viscous liquid (41.15 g). Into a flask, [VBMI]Cl (41.15 g), $NaBF_4$ (24.15 g, 0.22 mol), hydroquinone (0.10 g) and acetone (200 mL) were added under nitrogen atmosphere. The flask content was stirred at room temperature for 24 h with magnetic stirring, and the precipitate of sodium chloride (NaCl) was observed during the

process. The precipitate was removed, and the filtrate was concentrated *via* rotary evaporation at room temperature to remove about half of the solvent. The resultant viscous solution was added into iced diethyl ether to yield a white and waxy solid. The waxy solid was washed with iced diethyl ether (500 mL×2), and dried overnight at room temperature under vacuum to obtain the [VBMI][BF_4] monomer (44.56 g, 78% yield). Figure S1 shows the ^1H NMR spectrum of the [VBMI][BF_4] monomer.

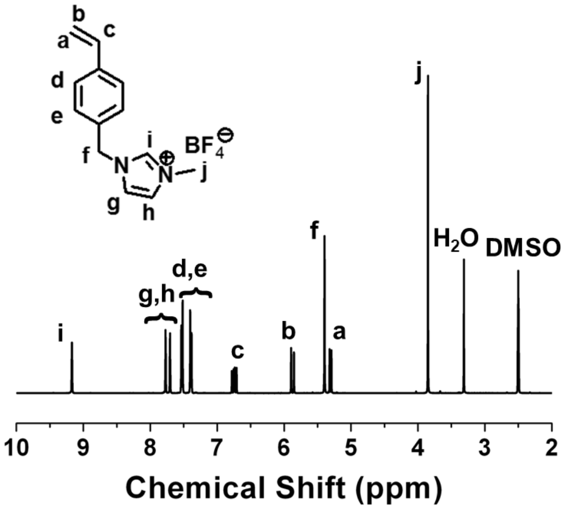

Figure S1. The ^1H NMR spectrum of [VBMI][BF_4].

2. Synthesis of PILs by solution RAFT polymerization

The homopolymer of PILs, P[VBMI][BF_4]-TTC, was prepared by solution RAFT polymerization in the 80/20 methanol/water mixture employing DDMAT as RAFT agent and AIBN as initiator under $[[VBMI][BF_4]]_0/[DDMAT]_0/[AIBN]_0$= 60~270:3:1. Herein, a typical RAFT polymerization under $[[VBMI][BF_4]]_0:[DDMAT]_0:[AIBN]_0$= 270:3:1 was introduced. Into a Schlenk flask with a magnetic bar, [VBMI][BF_4] (5.00 g, 17.54 mmol), DDMAT (71.2 mg, 0.195 mmol), and AIBN (10.7 mg, 0.065 mmol) dissolved in the methanol/water mixture (10.0 g, 80/20 w/w) were added. The flask content was initially degassed with nitrogen at 0 ℃, and then was immersed into a preheated oil bath at 70 ℃. After 6 h, the polymerization was quenched by rapid cooling upon immersion of the flask in iced water. To detect the monomer conversion, a given volume (0.3 mL) of the solution was filtrated twice with a 0.22 μm nylon filter, and then the filtrate was diluted with ethanol and analyzed by UV-vis analysis at 250 nm. To collect the synthesized PILs of P[VBMI][BF_4]-TTC, the flask content

was added dropwise into methanol to precipitate the PILs, and the collected precipitate was washed twice with methanol, and then washed with diethyl ether, and finally dried at 45 ℃ under vacuum to afford pale yellow powder of P[VBMI][BF_4]-TTC. By changing the molar ratio of [VBMI][BF_4]/DDMAT, P[VBMI][BF_4]-TTC with different polymerization degree (DP) as shown in Table 1 were prepared.

3. GPC analysis of PNIPAM$_{223}$

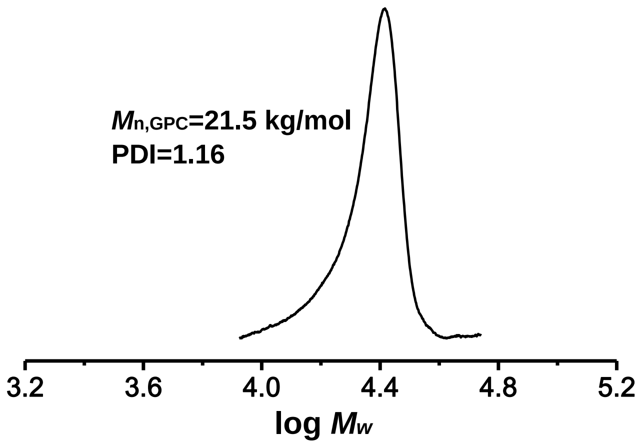

Figure S2. GPC curve of PNIPAM$_{223}$.

4. The temperature-dependent transmittance of the PNIPAM$_{223}$ and P[VBMI][BF_4]$_{76}$ mixture

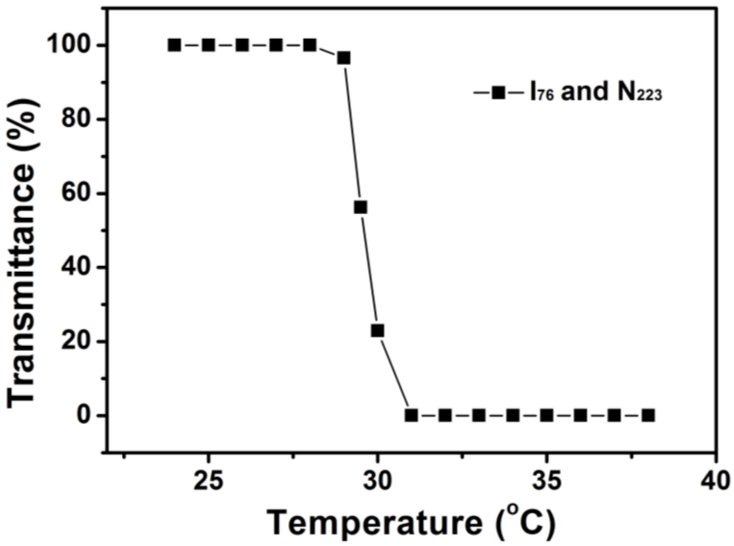

Figure S3. The temperature-dependent transmittance of 2 wt% aqueous solution containing equal molar of PNIPAM$_{223}$ and P[VBMI][BF_4]$_{76}$.

5. The temperature-dependent ^{1}H NMR spectra of $I_{76}N_{226}$ and $I_{76}N_{364}$

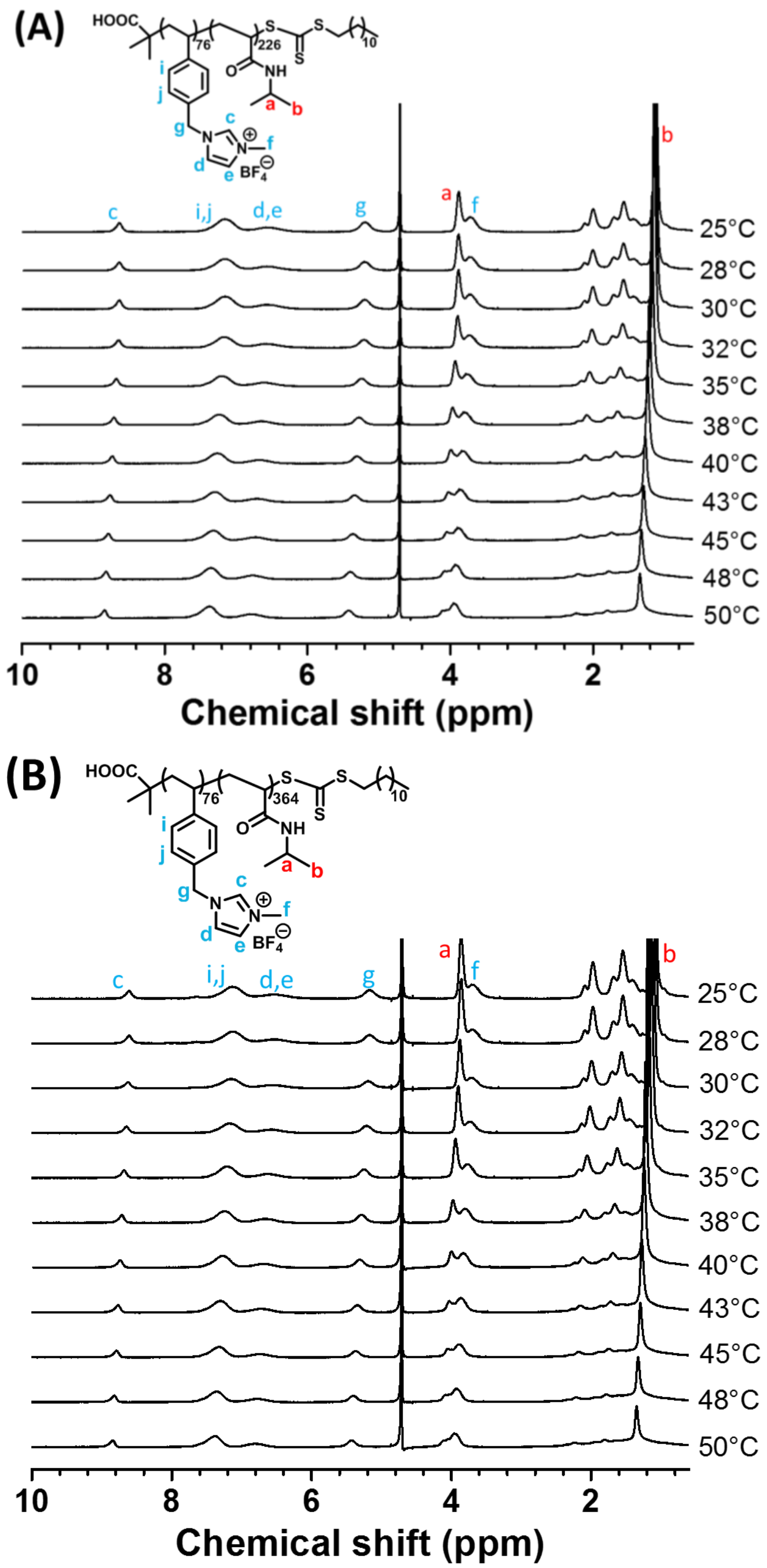

Figure S4. The temperature-dependent ^{1}H NMR spectra of $I_{76}N_{226}$ (A) and $I_{76}N_{364}$ (B) in D_2O.

6. DLS analysis

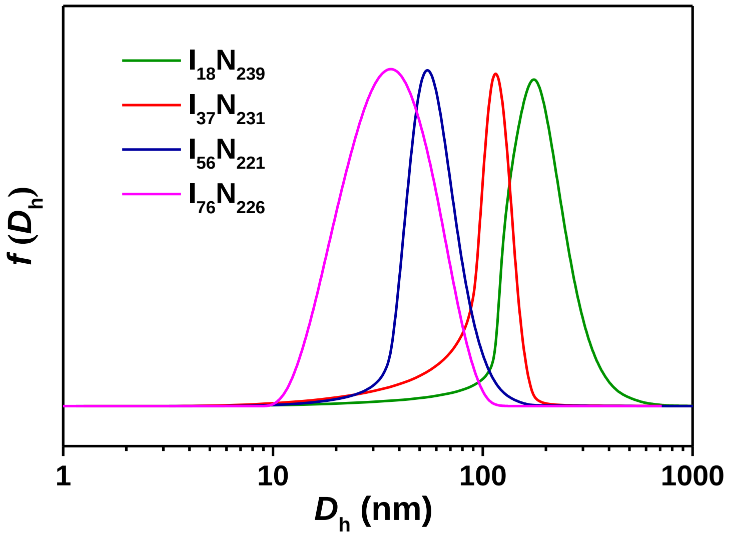

Figure S5. The hydrodynamic diameter distribution $f(D_h)$ of P[VBMI][BF_4]-*b*-PNIPAM aqueous solution at 50 ℃ (0.5% polymer aqueous solution, remove dust before measurement).

7. Equations

$$\text{conversion}=\left(1-\frac{6\times I_{6.25}}{I_{5.12}}\times\frac{[\text{trioxane}]_0}{[\text{NIPAM}]_0}\right)\times 100\% \quad (S1)$$

$$\text{conversion}=\frac{I_{1.05\sim 1.22}-6\times I_{6.00\sim 6.10}}{I_{1.05\sim 1.22}}\times 100\% \quad (S2)$$

$$M_{n,th}=\frac{[\text{monomer}]_0\times M_{\text{monomer}}}{[\text{RAFT}]_0}\times\text{conversion}+M_{\text{RAFT/macro-RAFT}} \quad (S3)$$

$$M_{n,NMR}(\text{PIL})=\text{DP}_{\text{IL}}\times M_{\text{IL}}+M_{\text{DDMAT}}=\frac{I_{3.87}}{3\times I_{0.88}}\times M_{\text{IL}}+M_{\text{DDMAT}} \quad (S4)$$

$$M_{n,NMR}=\text{DP}_{\text{PNIPAM}}\times M_{\text{NIPAM}}+M_{\text{PIL}}=\frac{I_{3.84}-3\times I_{9.1}}{I_{9.1}}\times\text{DP}_{\text{PIL}}\times M_{\text{NIPAM}}+M_{\text{PIL}} \quad (S5)$$

$$p=1-\frac{I_{1.17}}{I_{0(1.17)}} \quad (S6)$$
